# Gene signatures, immune infiltration, and drug sensitivity based on a comprehensive analysis of m6a RNA methylation regulators in cervical cancer

**DOI:** 10.1186/s12967-022-03600-7

**Published:** 2022-09-04

**Authors:** Xiaoqin Lu, Rui Li, Yanqi Ying, Wenyi Zhang, Wuliang Wang

**Affiliations:** Department of Obstetrics and Gynecology, the Second Affiliated Hospital of Zhengzhou University, 2nd, Jingba RoadHenan Province, Zhengzhou, 450053 China

**Keywords:** N^6^-dimethyladenosine, Immune infiltration, Drug sensitivity, Cervical cancer, ZC3H13, Rapamycin

## Abstract

**Background:**

Cervical cancer is the fourth most common cancer in women. N^6^-dimethyladenosine (m^6^A) mRNA methylation is closely associated with cervical cancer.

**Methods:**

Using TCGA database, we studied the expression and mutation of m^6^A-related genes in cervical squamous cell carcinoma and endocervical adenocarcinoma (CESC) and obtained genetic characteristics based on an m^6^A risk model and prognostic value of m^6^A. We studied the effects of the m^6^A risk score on immune features and genomic changes of patients with CESC, evaluated the sensitivity of patients with CESC to different small-molecule drugs based on the m^6^A risk score, and established a clinical prediction model.

**Results:**

Ten m^6^A-related genes were differentially expressed between CESC and normal tissues. High-risk patients had a low overall survival (OS) and significantly low immune scores but showed no significantly altered stromal scores. The tumor mutation burden (TMB) and tumor neoantigen levels significantly differed between the high- and low-risk groups. In the high-risk group, copy number variation (CNV) changes mainly led to gene amplification, while in the low-risk group, CNV changes primarily manifested as gene copy number deletions. ZC3H13 expression was low in CESC tissues. ZC3H13 knockdown promoted CESC cell proliferation, migration, and invasion, reducing the RNA methylation levels. Rapamycin suppressed the CESC cell proliferation, migration, and invasion abilities, increasing the m^6^A levels.

**Conclusion:**

m^6^A mRNA methylation is closely related to the occurrence, development, immune invasion, drug sensitivity, and prognosis of cervical cancer. The prognostic m^6^A feature model of m^6^A signature genes can accurately predict the OS of patients with CESC. Drugs targeting factors regulating m^6^A mRNA methylation might offer a good prospect for treating cervical cancer.

**Supplementary Information:**

The online version contains supplementary material available at 10.1186/s12967-022-03600-7.

## Introduction

Cervical cancer is the fourth most common cancer in women [1, 2]. More than 570,000 new cases of cervical cancer are diagnosed worldwide each year, with approximately 311,000 deaths [3]. Presently, at least 170 RNA modifications have been discovered in cervical cancer, for instance, N^6^-dimethyladenosine (m^6^A), inosine, pseudouridine, 5-methylcytidine (m5C), 5-hydroxymethylcytidine (hm5C), and N1-methyladenosine (m1A). Furthermore, most RNA species contain at least one chemical modification and are widely associated with physiopathological processes [4–7]. m^6^A is one of the most abundant mRNA modifications [8, 9]. m^6^A modifies approximately 0.1–0.4% of all adenosines in RNA, accounting for ~ 50% of the total methylated ribonucleotides [10]. m^6^A is a dynamic and reversible epigenetic modification. The cellular m^6^A status is regulated by groups of proteins called m^6^A methyltransferases (“writers”), m^6^A demethylases (“erasers”), and m^6^A-binding proteins (“readers”), which add, remove, or recognize m^6^A-modified sites, respectively, modulating the stability, splicing, intracellular distribution, and translational changes of mRNA while affecting certain biological processes [11–14]. As a potential tumor biomarker, m^6^A plays a key role in various biological processes and malignancies [15]. Changes in m^6^A-modifying enzyme levels affect the expression of downstream oncogenes or tumor suppressor genes by altering mRNA methylation [16]. Furthermore, epigenetic markers potentially serve as diagnostic, prognostic, and predictive biomarkers and might be used as novel targets for cancer precision medicine [17–20].

Here, we studied the expression and mutation of m^6^A-related genes in patients with cervical squamous cell carcinoma and endocervical adenocarcinoma (CESC), obtained the gene characteristics of patients with CESC based on an m^6^A-risk model, and constructed a prognostic m^6^A feature model based on m^6^A signature genes. The effects of the m^6^A risk score on biological function, immune characteristics, and genomic changes of patients with CESC were analyzed. The sensitivity of patients with CESC to different small molecule drugs was evaluated based on the m^6^A risk score, and a clinical prediction model was constructed. Subsequently, we studied the expression and function of ZC3H13 in cervical cancer tissues according to the screening results. We selected rapamycin to study the effects of the screened drug on m^6^A and cervical cancer phenotype. Through this research, we attempted to study the gene signatures, immune infiltration, and drug sensitivity based on a comprehensive analysis of m^6^A RNA methylation regulators in cervical cancer.

## Materials and methods

### Data

Data were downloaded from the TCGA GDC website (https://portal.gdc.cancer.gov/), and the cervical squamous carcinoma and adenocarcinoma (cervical squamous cell carcinoma and endocervical adenocarcinoma, CESC) expression profile data (FPKM results) of gene expression sequencing in patients were obtained. The FPKM values were converted into the TPM values as follows:

$${\mathrm{TPM}}_{i}=\left(\frac{{\mathrm{FPKM}}_{i}}{{\sum }_{j}{\mathrm{FPKM}}_{\mathrm{j}}}\right)\bullet {10}^{6}$$

Clinical data of corresponding patients, including age, TNM stage, and survival prognosis, were downloaded. After excluding patients with CESC with missing clinical information, data regarding 279 tumor tissues and three paracancer tissues were obtained. The copy number variation (CNV) of somatic cells of patients with CESC was downloaded. RCircos package was used to map the gene CNV in 23 pairs of chromosomes [21]. “Masked mutation” was selected as the somatic mutation data, and R’s MAfTools package was used to visualize somatic mutations. The tumor mutation burden (TMB) of each patient was obtained [22]. The sequencing results of 19 cervical tissues were downloaded from the GTEx database and converted into TPM values.

In addition, gene expression data of the GSE52903 chip, clinicopathological features, and prognostic information of patients were downloaded from samples in the GEO database, including 55 tumor tissue and 17 normal cervical tissue samples [23]. Among them, all chip data samples were from *Homo Sapiens*, and the platform was mainly based on the GPL6244 [Hugene-1_0-ST] Affymetrix Human Gene 1.0 ST Array [Transcript (Gene) version]. Next, TCGA data, GTEx data, and GEO chip data were combined, and the limma and sva packages of R were used for homogenization and batch removal [24, 25]. The total process is composed of two steps. The first is data consolidation; only common genes and corresponding expression values are retained in the three data sets. The second step involves removing the batch effects. The first step adopts the merge function in R to merge the three data sets. The second step uses the combat function in the SVA package to remove the batches. The demographic and clinical characteristics of the cervical cancer patients are shown in Additional file 1 materials.

### Risk model construction based on m^6^A-related genes

To analyze the expression of m^6^A-related genes in cervical cancer, we first analyzed the differential expression of m^6^A-related genes in cervical cancer and normal tissues, the correlation of gene expression, and its impact on the prognosis of patients with CESC. Subsequently, expression data of patients with CESC were pooled using TCGA-CESC and GEO data. m^6^A-related genes were included in the model, and the least absolute shrinkage and selection operator (LASSO) algorithm was used to analyze the dimension reduction and obtain characteristic genes associated with prognosis. A risk score formula was established using the penalty coefficient weighted normalized gene expression values obtained by LASSO Cox analysis. The patients were divided into high- and low-risk groups based on the median risk score.

$$\mathrm{risk}Score = \sum_{i}Coefficient \left({hub gene}_{i}\right)*mRNA Expression (hub {gene}_{i})$$

### Gene set enrichment analysis (GSEA)

Gene Ontology (GO) analysis is a common method for large-scale functional enrichment studies that includes three categories: biological process (BP), molecular function (MF), and cellular component (CC). KEGG is a widely used database that stores information regarding genomes, biological pathways, diseases, and drugs. R's clusterProfiler package was used for GO annotation analysis and KEGG pathway enrichment analysis of signature genes. A value of FDR < 0.05 was considered statistically significant [26].

To study the differences in BPs between different subgroups, we performed GSEA based on the gene expression profile dataset of patients with CESC. GSEA is a computational method to analyze whether a specific gene set has statistically significant differences between two biological states and is usually used to estimate changes in pathways and BP activity among samples [27]. The “h.all.v7.2.symbols.gmt” gene set was downloaded from MSigDB for GSEA analysis [27]. An adjusted P-value of less than 0.05 was considered statistically significant.

### Assessment of patients’ biological characteristics between risk groups

We further analyzed the correlation between different subgroups and some biologically related processes using the GSVA method [27]. Mariathasan et al. constructed a gene set for storing genes related to some BPs, including (1) immune checkpoint; (2) antigen processing; (3) characteristics of CD8^+^ T cells; (4) epithelial-mesenchymal transformation (EMT) markers, including EMT1, EMT2, and EMT3; (5) angiogenesis; (7) characteristics of TGF-β response in pan-FTBRs; (8) WNT characteristics; (9) DNA damage repair; (10) mismatch repair; (11) nucleotide excision repair; (12) DNA replication; and (13) antigen handling and presentation [28–30]. Gene sets corresponding to different biological characteristics were downloaded to calculate the corresponding enrichment scores of patients and to compare the differences between two groups.

### Analysis of m.^6^A-related differentially expressed genes (DEGs)

To identify genes associated with the m^6^A risk model, THE limma package of R was used to analyze DEGs between high and low subgroups in patients with CESC, and the DEGs with significant differences were defined as genes with absolute log (Fold change) > 0.5 and FDR < 0.05 [24]. Hierarchical clustering was used to divide the tumors into different gene groups based on the Euclidean distance of differential gene expression and named them Geneclusters A, B, and C. Among them, R's ConsensusClusterPlus package was used to determine the number of clusters in the dataset and was repeated 1000 times to ensure the stability of classification [31]. Meanwhile, based on the expression changes of specific genes, they were divided into signature-A and -B gene groups.

### Calculation of dimensionality reduction and m^6^A score

First, unsupervised clustering was used to classify TCGA data of patients according to DEG values. m^6^A signature-A and -B gene groups were reduced in dimension according to the Boruta algorithm. The principal component PC1 was extracted by the principal component analysis (PCA) algorithm as the A score. Finally, we applied a method similar to the gene expression grade index to define each patient’s immune checkpoint inhibitor (ICI) score:

$$m6A score = \sum_{i}PC1A- \sum_{i}PC1B$$

### Identification and correlation analysis of tumor immune infiltrating cells

To further quantify the proportion of different immune cells in CESC samples, the CIBERSORT algorithm, and LM22 gene set were used to investigate the phenotypes of 22 human immune cells (including B cells, T cells, and natural killer cells) in the tumor microenvironment (TME) [32]. Macrophages are highly sensitive and specific. CIBERSORT is a deconvolution algorithm that uses a set of reference gene expression values (with 547 characteristic genes) considered to be the minimum representative of each cell type. Based on these values, the proportion of cell types can be inferred in data from a large tumor sample with mixed cell types.

At the same time, R's ESTIMATE package was used to assess tumor immune activity [33]. ESTIMATE an immune score for each tumor sample obtained by quantifying the immune activity (level of immune invasion) in the tumor sample based on its gene expression profile. Then, differences in the characteristics of immune infiltration in patients with CESC between the high- and low-risk groups were obtained.

### Analysis of CNV

To analyze the changes in the copy number in different risk score groups of TCGA-patients with CESC, the TCGAbiolinks package of R was used to download the masked copy number segment data of patients. GISTIC 2.0 analysis of the downloaded CNV fragments was performed by GenePattern5. During analysis, except for a few parameters (e.g., the confidence is 0.99; Excluding X chromosomes prior to analysis), GISTIC 2.0 analysis was used with the default settings. Finally, R's MAfTools package was used to visually display the results of the GISTIC 2.0 analysis.

### Sensitivity analysis of anticancer drugs

Genomics of Drug Sensitivity in Cancer (GDSC; https://www.cancerrxgene.org/), which is used for molecular cancer therapy and mutation, was used to explore public databases [34]. R's pRRophetic package was used to download cell line gene mutation data and IC_50_ values of different anticancer drugs to analyze the correlation between patients with high- and low-risk scores and sensitivity to different anticancer drugs [35].

### Building a clinical prediction model based on the m^6^A risk model

To prove that the risk score combined with the clinicopathological features can help in the personalized assessment of patient prognosis, univariate and multivariate Cox analyses were conducted to analyze the ability of the risk score combined with clinicopathological features to predict the overall survival (OS). Then, a nomogram was constructed by incorporating the risk scoring model and clinicopathological features into the model. To quantify the differentiated performance, Harrell’s Consistency index (C-index) was measured. A calibration curve was generated to evaluate the performance of the rosette by comparing the predicted values of the rosette with the actual observed survival rates.

### Human tissue samples

Ten cervical cancer tissues and ten normal cervical tissues were obtained from patients with cervical cancer who underwent ovariectomy prior to chemotherapy and radiotherapy. The resected cervical cancer tissues and normal tissues were immediately stored at − 80 °C for further study. All patients signed informed consent forms. This study was performed in accordance with the Helsinki Declaration and approved by the ethics committee of the Second Affiliated Hospital of Zhengzhou University.

### Cell lines and culture conditions

The human cervical cancer cell lines HeLa and SiHa were purchased from Procell Life Science & Technology Co., Ltd. (Wuhan, P.R. China). To prepare the complete growth medium, the cell culture media were supplemented with fetal bovine serum (Gibco, Grand Island, USA) at a final concentration of 10%.

### RNA extraction

Total RNAs of human cells were extracted using the Trizol reagent (Invitrogen, Carlsbad, CA, USA) according to the manufacturer’s instructions and treated with RQ1 DNase (Promega, Madison, WI, USA) to remove DNA. The quality and quantity of purified RNA were determined by measuring the absorbance at 260 nm and 280 nm (A260 and A280, respectively) using a SmartSpec Plus Spectrophotometer (Bio-Rad Laboratories, Inc., Hercules, CA, USA). RNA integrity was further verified by electrophoresis using a 1.5% agarose gel. All RNA samples were stored at -80 °C for future analysis. Reverse transcription reactions were carried out using the ReverTra Ace qPCR RT Kit (TOYOBO Life Science, Shanghai, P.R. China), according to the manufacturer’s instructions.

### Quantitative real-time PCR (qRT-PCR)

Expression levels of the ZC3H13 gene were detected by qRT-PCR. The human (species) ACTB gene was used as a control. Specific primers were designed based on cDNA sequences. Primer sequences of ZC3H13 were as follows: 5′- ACATTCATTAGGCTCTGGTGC -3′ forward), 5′—TTCTCCTCATCCTGTTGGTCC—3′ (reverse). qRT-PCR was performed on a Bio-Rad S1000 with Bestar SYBR GreenRT-PCR Master Mix (TOYOBO). PCR conditions consisted of denaturing at 95 °C for 1 min, and 40 cycles of denaturing at 95 °C for 15 s followed by annealing and extension at 60 °C for 30 s. The relative gene expression was calculated using the Livak and Schmittgen 2^−ΔΔCt^ method (Livak and Schmittgen 2001), normalized with the reference gene actin. PCR amplifications were performed in triplicate for each sample.

### Immunohistochemical analysis

The tissue slides (4-μm-thick sections) were initially treated for deparaffinization, rehydration, and antigen-retrieval using 3% H_2_O_2_. The sections were incubated with anti-ZC3H13 (Bioss, P.R. China) and then with horseradish peroxidase (HRP)-labeled IgG secondary antibodies (Beyotime Institute of Biotechnology, Shanghai, P.R. China). Fields from each slide were examined and photographed using a light microscope (Olympus, Japan).

### Western blot analysis

Cells were lysed in a radioimmunoprecipitation assay buffer containing 1 nM phenylmethanesulfonyl fluoride, and protein concentrations were determined using a bicinchoninic acid (BCA) protein assay kit (Biosharp, Guangzhou, China) according to the manufacturer’s instructions. Protein samples (40 μg) of each group were boiled with 6 × sodium dodecyl sulfate loading buffer for 10 min before electrophoresis on 12% sodium dodecyl sulfate–polyacrylamide gel electrophoresis gels. The resolved proteins were electrotransferred onto polyvinylidene difluoride membranes (Millipore, Billerica, MA, USA) in a transferring buffer (25 mM Tris, 0.2 M glycine, and 25% methanol). After blocking with 5% skimmed milk, the membranes were incubated with anti-ZC3H13 (Bioss, P.R. China) and anti-β-actin antibodies (Bioss, P.R. China), followed by incubation with appropriate HRP-conjugated secondary antibodies (Abcam, England).

### Cell transfection

ZC3H13-siRNA (5′-GAAGACAUCUGCAGUAUCU-3′, antisense 5′-AGAUACUGCAGAUGUCUUC-3′) was synthesized by GeneCreate Bioengineering Co., Ltd. Gene Create (Wuhan, P.R. China). According to the manufacturer’s instructions, Lipofectamine 2000 (Invitrogen, USA) was used for transfecting siRNAs into HeLa and SiHa cells.

### CCK8 assay

Cells were digested with trypsin and inoculated into 96-well plates. The culture plate was placed in a 5% CO_2_ incubator for 0, 24, and 48 h at 37℃. Next, rapamycin (GlpBio, USA) and/or FBS (Gibco, USA) were added. Then, the CCK-8 (Solarbio, Beijing, China) solution was added, and the samples were incubated for 0.5 h. The absorbance of each well was measured at 450 nm.

### Transwell chamber assay

The cell invasion ability was evaluated using Transwell chambers precoated with Matrigel. A total of 1 × 10^4^ cells were inoculated into the top chamber and incubated at 37 °C with 5% CO_2_ for 48 h. After cells on top of the filter were removed, cells on the bottom of the filter were fixed in 4% paraformaldehyde and stained with 1% crystal violet (Beyotime, P.R. China). The invading cells were counted under a microscope.

### Wound-healing assay

Approximately 5 × 10^5^ HeLa cells were seeded in 6-well plates. The cells were washed with PBS three times, and fresh medium was added. Cells were cultured in a 37 ℃ 5% CO_2_ incubator, photographed, and recorded under the microscope at 0, 24, and 48 h.

### m^6^A ELISA

The total RNA extracted was detected by EpiQuik ™ m^6^A RNA Methylation Quantification Kit (Epigentek, USA), following the manufacturer’s instructions.

### Statistical analysis

All data processing and analysis were completed in R (version 3.6.2). To compare two groups of continuous variables, the statistical significance of the normally distributed variables was estimated using the independent student’s t-test, and the differences between non-normally distributed variables were analyzed using the Mann–Whitney U test (i.e., Wilcoxon rank-sum test). The Chi-square test or Fisher’s exact test was used to compare and analyze the statistical significance between two groups of categorical variables. The correlation coefficients of different genes were calculated using Pearson correlation analysis. R’s survival package was used for survival analysis, the Kaplan–Meier survival curve was used to highlight the survival difference, and the log-rank test was used to evaluate the significance of the survival time difference between two groups [36]. R’s pROC package was used to plot the receiver operating characteristic (ROC) curve and calculate the area under the curve (AUC) to assess the accuracy of the risk score in estimating prognosis. Univariate and multivariate Cox analyses were used to determine independent prognostic factors [37]. All statistical P-values were bilateral, and P < 0.05 was considered statistically significant.

## Results

**Fig. 1:**
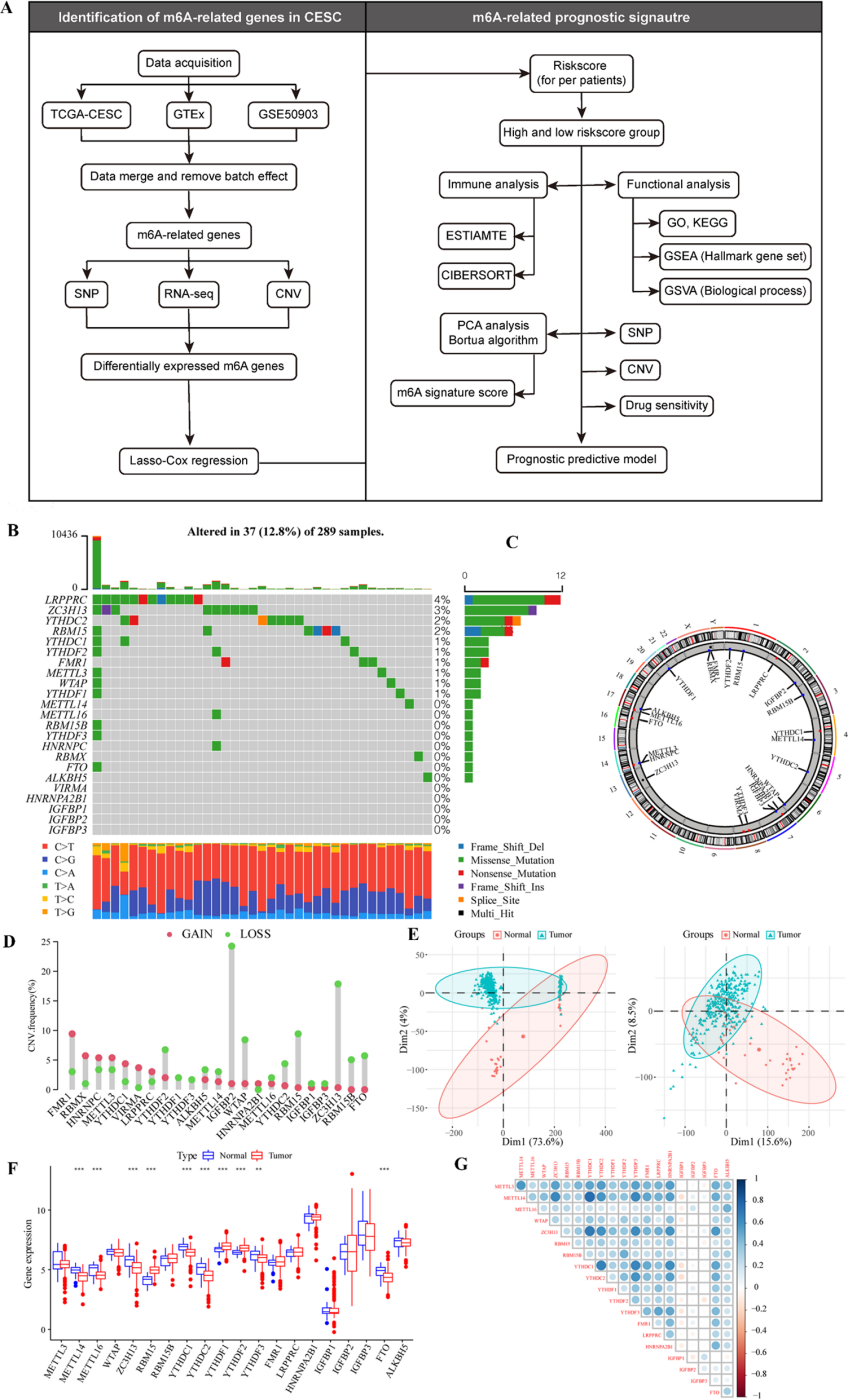
Overall expression of m^6^A-related genes in patients with CESC. **A** Workflow of the study. B Mutation map of m^6^A-related genes in TCGA-patients with CESC. Samples were sequenced according to the burden of nonsynonymous mutations in somatic cells, and genes were sequenced according to the mutation frequency. Different colors indicate different mutation types. The section above the legend shows the mutation load; **C** CNV differential changes and locations of m^6^A-related genes on 23 chromosomes of patients with TCGA-CESC; **D** change frequency of CNV level of m^6^A-related genes, where red represents CNV level amplification and green represents CNV level deletion; **E** PCA showed differences between cervical and normal cervical tissues before and after combination and batch effect removal in TCGA, GTEx, and GEO datasets. The left panel shows the result before batch effect removal, and the right shows the result after batch effect removal. **F** Differentially expressed m^6^A-related genes in cervical cancer and normal cervical tissue; **G** expression correlation analysis of m^6^A-related genes in CESC

A flow chart of the study is shown in Fig. 1A.

### Expression and mutation profile of m^6^A-related genes in patients with CESC

To analyze the overall expression of m^6^A-related genes in patients with CESC, we analyzed the genomic mutations and mRNA expression, including single nucleotide polymorphism, CNV, and gene expression level. SNP analysis showed that 37 of 289 samples had single nucleotide mutations in m^6^A-related genes (Fig. 1B). Moreover, studies on the change frequency of CNV showed that the changes of m^6^A-related genes in CNV levels were widespread in patients with CESC, and most were focused on the loss of copy number. Fig.  1C and D show the position and frequency of CNV changes on chromosomes, respectively.

Next, we comprehensively analyzed the expression of m^6^A-related genes in cervical cancer and cervical tissue in the TCGA database, GTEx database, and GSE52903 dataset, and batch effects were removed. PCA results showed significant differences in overall expression levels between tumor samples and normal tissues after batch removal (Fig. 1E). We focused on m^6^A-related genes and compared the differences of m^6^A-related genes between the two groups by the Wilcoxon rank-sum test. Differential analysis results showed that many m^6^A-related genes were differentially expressed between cervical cancer tissues and normal tissues, including METTL14, METTL16, ZC3H13, YTHDC1, and YTHDC2 (Fig. 1F). After further correlation analysis, a heat map was constructed, which showed the correlation of m^6^A-related gene expression. Most genes showed a positive correlation in CESC tissues (Fig. 1G).

**Fig. 2:**
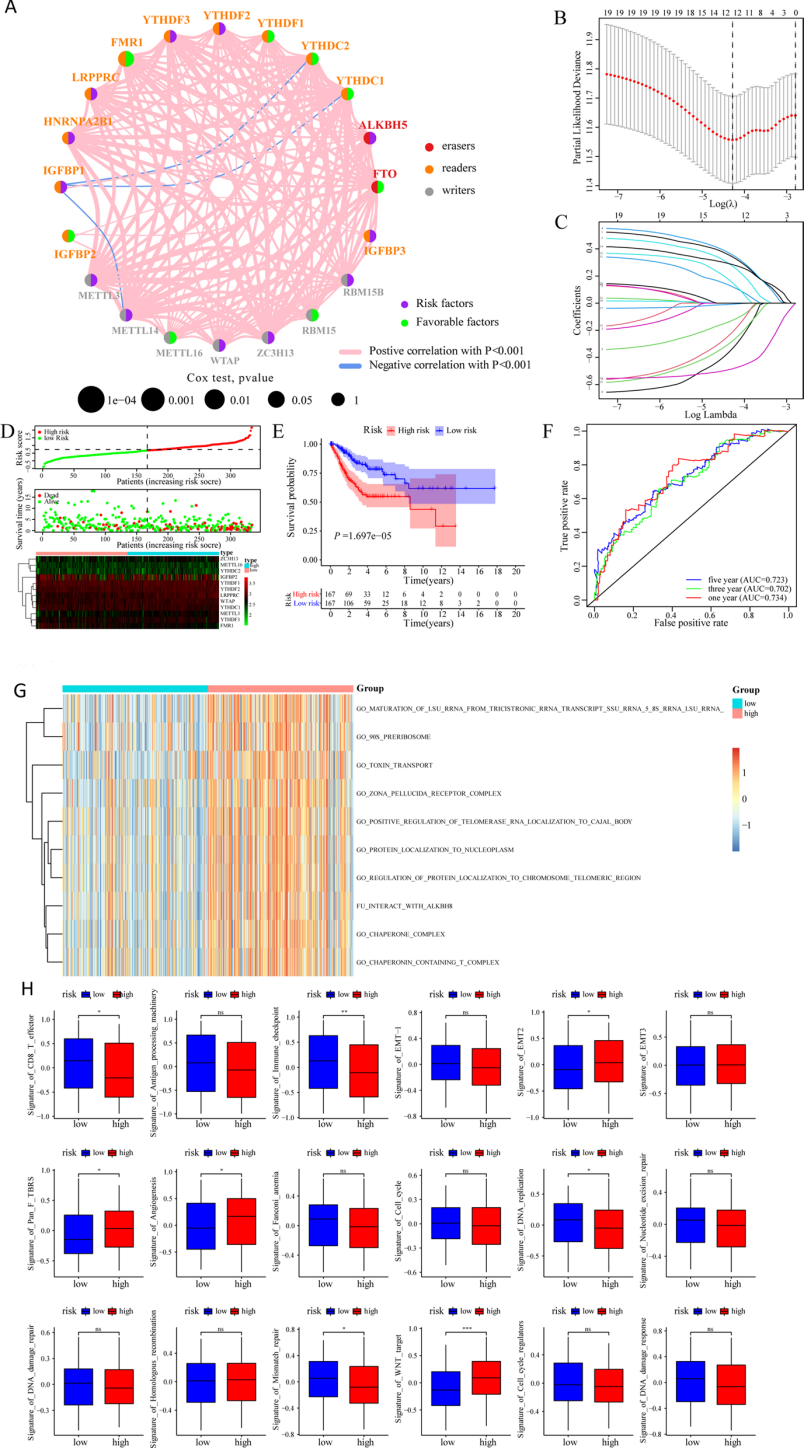
Construction of m^6^A risk scoring model and influence of m^6^A risk model on different biological characteristics. **A** Expression and interaction of m^6^A-related genes in patients with CESC. The size of each cell represents the impact of each gene on patient survival, which was analyzed using the logarithmic rank test. The color of one half of the circles represents the m^6^A-related gene group, and the other half represents the effect on prognosis. The m^6^A-related genes were grouped as erasers (red), readers (orange), and writers (gray). Meanwhile, in terms of the impact on prognosis, purple represents risk factors, and green represents protective factors. The lines connecting the m^6^A-related genes represent interactions between genes. The thickness of the line represents the correlation intensity estimated by the Spearman correlation analysis. The positive correlation is represented by red, and the negative correlation is represented by blue. **B**, **C** LASSO Cox analysis identified 12 genes most associated with TCGA data set OS. **D** Distribution of risk scores for patients with CESC, patient survival, and heat maps of characteristic gene expression; **E** Kaplan–Meier curve analysis to assess the effect of risk score on overall survival in patients with CESC; **F** ROC curve analysis of time dependence of risk score. **G** GSVA analysis of patients in the high- and low-risk subgroups based on gene expression data of patients with CESC. Heat maps were used to demonstrate the relevant pathways with significant differential enrichment. **H** Enrichment of different pathway characteristics (immune-related characteristics, mismatch-related characteristics, and matrix-related characteristics) in the high- and low-risk score subgroups, where thick lines represent the median. The bottom and top of the box are the 25th and 75th percentiles (interquartile spacing) (*P < 0.05, **P < 0.01, ***P < 0.001)

We further selected and analyzed the influence of m^6^A-related genes on the prognosis of patients with CESC based on the TCGA and GEO databases. The gene network depicted a comprehensive picture of m^6^A-related gene interactions, source grouping, and their impact on OS in patients with CESC (Fig. 2A).

Subsequently, we constructed a risk scoring system based on the expression of m^6^A-related genes to quantitatively evaluate the impact of m^6^A-related genes on the prognosis of each patient with CESC. First, 20 m^6^A-related genes were included in the LASSO Cox analysis, and 12 genes with the best prognostic value were selected and identified (Fig. 2B, C). Simultaneously, based on the penalty coefficient of important characteristic genes calculated by LASSO Cox analysis, the gene expression was multiplied and added to the corresponding coefficient to establish the risk score, and the final risk score of each sample was calculated. The significance of the LASSO Cox analysis here was to eliminate the multicollinearity between genes (resulting from the use of LASSO), and ultimately, to ensure that the genes included in the multivariate Cox regression are independent. The distribution of risk score, survival status, and expression patterns of characteristic genes were shown in Fig. 2D. Kaplan–Meier analysis showed that the OS of patients with high-risk scores was relatively poor (log-rank P < 0.001; Fig. 2E). The time-dependent ROC analysis of the risk score showed that the risk score had a good predictive ability for the OS of patients with CESC, and the AUC of 1-, 3-, and 5-year OS were 0.734 (0.642–0.825), 0.702 (0.648–0.797), and 0.723 (0.665–0.829), respectively (Fig. 2F).

### Influence of m^6^A risk score on the biological function of patients with CESC

We then divided patients into high- and low-risk groups based on the median m^6^A risk score for patients with CESC and assessed the changes in biological function between the two groups. To analyze the variation in different pathways in patients with CESC, the enrichment scores of patients with CESC were analyzed using the GSVA method, and the related signaling pathways with significant differential expression in the high- and low-risk patients were shown through heat maps (Fig. 2G). In addition, the analysis revealed significant differences in the enrichment of related biological pathways, such as CD8^+^ T cell effectors, immune checkpoints, EMT pathways, and angiogenesis, between high- and low-risk patients (P < 0.05; Fig. 2H).

### Construction of genetic characteristics of patients with CESC based on the m^6^A risk model

**Fig. 3:**
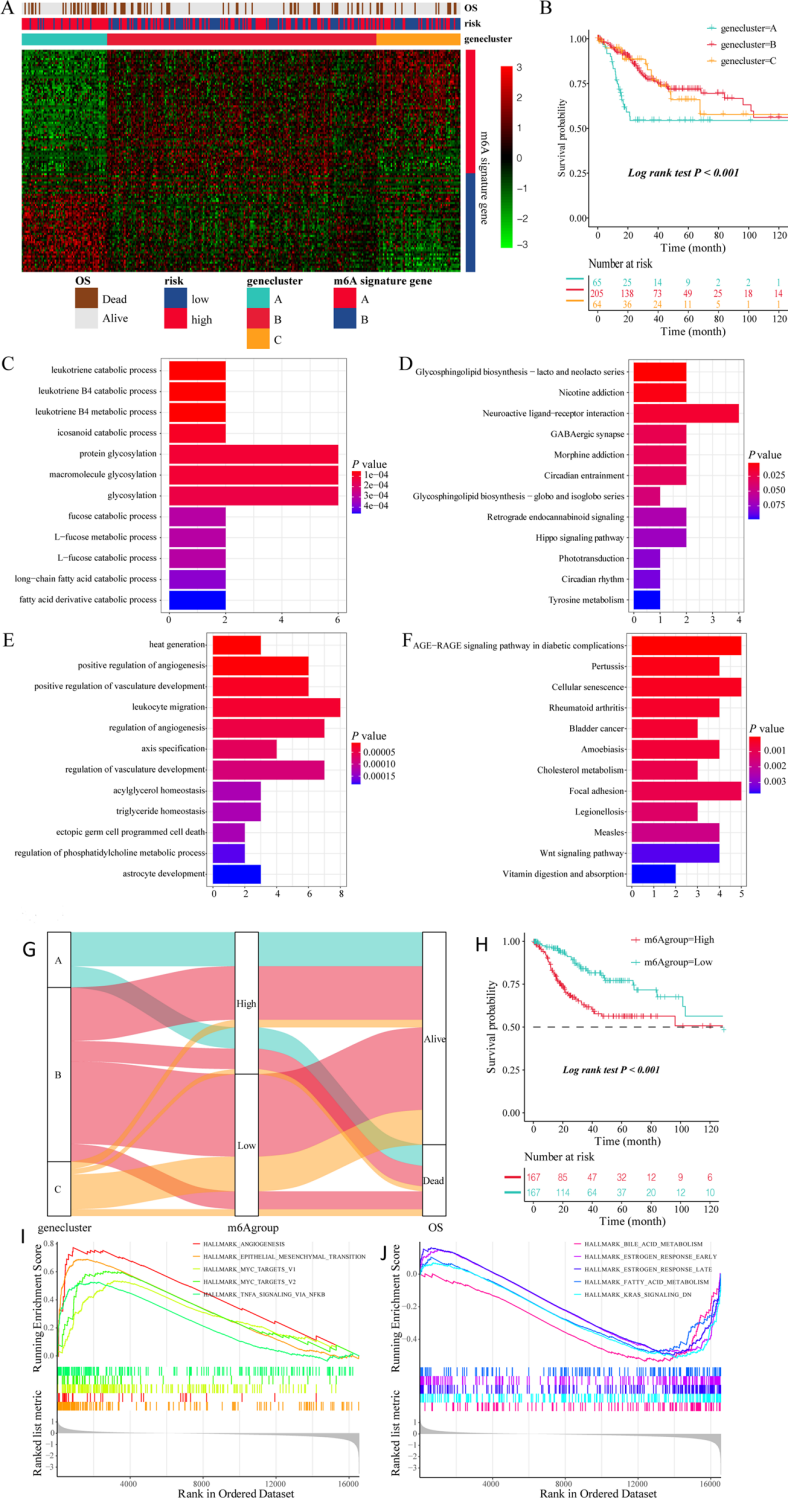
Construction and functional annotation of the m^6^A gene characteristic model, construction of prognostic m^6^A feature model, and regulation of BPs in patients with CESC. **A** Based on the expression characteristics of DEGs between high and low m^6^A risk scores, unsupervised analysis and hierarchical clustering were performed to classify patients into three categories: Geneclusters A, B, and C, and DEGs were divided into signature -A and -B gene sets according to their expression changes. **B** Survival analysis showed significant differences in prognosis among the different Genecluster groups, and Genecluster A had the worst prognosis. **C** GO analysis showed that signature gene-A was involved in leukotriene catabolic processes, leukotriene B4 catabolic processes, and leukotriene B4 metabolic processes. **D** KEGG analysis showed that the signature-A gene set is closely related to glycosphingolipid biosynthesis-lacto and neolacto series, nicotine addiction, and other pathways. **E** GO analysis showed that the signature-B gene set was involved in heat generation, positive regulation of angiogenesis, and positive regulation of vasculature development. **F** KEGG enrichment analysis showed that signature-B and age-range signaling pathways in diabetic complications, pertussis, cellular senescence, and other pathways are closely related. **G** Sankey plots show correlations between the different gene clusters (Geneclusters A, B, and C, prognostic m^6^A traits (m^6^A group), and patient prognostic status (OS). **H** Survival analysis showed that the prognostic m^6^A feature model could better predict the OS of patients with CESC (log-rank P < 0.001). **I**, **J** GSEA for high- and low-risk patients. A signature gene set was downloaded from the MSigDB database, with 1,000 replicates per run. Epithelial-mesenchymal transition, angiogenesis, and Myc targets V1 were mainly enriched in high-risk groups, while bile acid metabolism, KRAS signaling DN, estrogen response late, and other pathways were significantly enriched in low-risk patients

To determine the underlying biological characteristics of different m^6^A-associated phenotypes, the LIMMA package was used to analyze differential genes between different risk models in the dataset and yielded 207 DEGs. The DEGs are shown in Additional file 2 materials. Subsequently, based on the DEGs, patients with CESC were divided into three different subtypes named Geneclusters A, B, and C (Fig. 3A) using unsupervised clustering. According to the expression and correlation of DEGs between different groups, genes were divided into m^6^A signature gene-A and B groups. At the same time, survival analysis results showed significant differences in the prognosis of patients with three different gene subtypes. Patients in Genecluster A had the worst prognosis (log-rank P < 0.001; Fig. 3B).

DEGs were divided into two groups, m^6^A signature-A and m^6^A signature-B, according to the correlation of gene expression. m^6^A signature-A gene set had 101 DEGs, and m^6^A signature-B gene set included 106 DEGs. To explore differences in biological functions between the two groups of genes, functional enrichment analysis was performed (Tables 1 and 2). GO and KEGG enrichment analysis showed that signature-A and -B gene sets showed different unique BPs. Signature-A gene set involved leukotriene catabolic processes, leukotriene B4 catabolic processes, leukotriene B4 metabolic processes, glycosphingolipid biosynthesis-lacto and neolacto series, nicotine addiction, and other pathways (Fig. 3C, D). In contrast, the gene set of overexpressed signature gene-B mainly showed heat generation, positive regulation of angiogenesis, positive regulation of vasculature development, AGE-RAGE signaling pathway in diabetic complications, pertussis, cellular senescence, and other pathways (Fig. 3E, F). The pathways are shown in Additional file 3 materials.

**Table 1:** GO analysis of m6A signature genes

ONTOLOGY	ID	Description	Count	P value	Gene
m6A Signature gene-A					
BP	GO:0036100	leukotriene catabolic process	2	5.09E− 05	CYP4F12/CYP4F3
BP	GO:0036101	leukotriene B4 catabolic process	2	5.09E− 05	CYP4F12/CYP4F3
BP	GO:0036102	leukotriene B4 metabolic process	2	5.09E− 05	CYP4F12/CYP4F3
CC	GO:0016324	apical plasma membrane	7	3.52E− 05	CEACAM5/CEACAM6/MUC20/CEACAM7/CYP4F12/SCNN1B/DUOX1
CC	GO:0045177	apical part of cell	7	0.000115	CEACAM5/CEACAM6/MUC20/CEACAM7/CYP4F12/SCNN1B/DUOX1
CC	GO:1902711	GABA-A receptor complex	2	0.00136	GABRP/GABRE
MF	GO:0070330	aromatase activity	3	1.01E− 05	CYP4X1/CYP4F12/CYP4B1
MF	GO:0050051	leukotriene-B4 20-monooxygenase activity	2	5.67E− 05	CYP4F12/CYP4F3
MF	GO:0020037	heme binding	5	6.14E− 05	CYP4X1/CYP4F12/CYP4F3/DUOX1/CYP4B1
m6A Signature gene-B					
BP	GO:0031649	heat generation	3	9.92E− 06	APLN/IL1B/IL1A
BP	GO:0045766	positive regulation of angiogenesis	6	1.17E− 05	CXCL8/DLL1/ANGPTL4/IL1B/ITGA5/IL1A
BP	GO:1904018	positive regulation of vasculature development	6	2.31E− 05	CXCL8/DLL1/ANGPTL4/IL1B/ITGA5/IL1A
MF	GO:0005342	organic acid transmembrane transporter activity	4	0.000661	SLC19A1/SLC6A11/SLC7A5/SLC16A1
MF	GO:0046943	carboxylic acid transmembrane transporter activity	4	0.000661	SLC19A1/SLC6A11/SLC7A5/SLC16A1
MF	GO:0005149	interleukin-1 receptor binding	2	0.000775	IL1B/IL1A

**Table 2:** KEGG analysis of m6A signature genes

ID	Description	Count	P value	Gene
**m6A Signature gene-A**				
hsa00601	Glycosphingolipid biosynthesis—lacto and neolacto series	2	0.00259	FUT2/FUT6
hsa05033	Nicotine addiction	2	0.005628	GABRP/ GABRE
hsa04080	Neuroactive ligand-receptor interaction	4	0.014459	GABRP/NPW/NMU/GABRE
hsa04727	GABAergic synapse	2	0.025976	GABRP/GABRE
hsa05032	Morphine addiction	2	0.02707	GABRP/GABRE
**m6A Signature gene-B**				
hsa04933	AGE-RAGE signaling pathway in diabetic complications	5	3.41E-05	CXCL8/CCND1/COL4A6/IL1B/ IL1A
hsa05133	Pertussis	4	0.000185	CXCL8/IL1B/ITGA5/IL1A
hsa04218	Cellular senescence	5	0.000281	CXCL8/CCND1/CCND2/MYC/IL1A
hsa05323	Rheumatoid arthritis	4	0.000403	CXCL2/CXCL8/IL1B/IL1A
hsa05219	Bladder cancer	3	0.00049	CXCL8/CCND1/MYC

### Construction of prognostic m^6^A feature model based on m^6^A signature gene

To better predict the impact of m^6^A characteristics on patient prognosis, we constructed a new prognostic-related risk scoring system. According to the expression of m^6^A Signature-A and -B gene sets in patients with CESC, the corresponding PCA1 of m^6^A signature-A and -B gene sets in each patient was calculated by PCA, and the corresponding m^6^A score of each patient was obtained by subtraction, which was named the m^6^A group. Similarly, patients were divided into high- and low-risk groups based on the median prognostic model scores. The Sankey diagram showed the corresponding gene group (Geneclusters A, B, and C) of each patient with CESC, the prognosis model m^6^A group, and the corresponding relationship among the survival statuses of patients (Fig. 3G). Simultaneously, survival analysis showed that the prognostic score model could accurately predict the OS of patients with CESC (log-rank P < 0.001; Fig. 3H).

Subsequently, we analyzed the effect of the high- and low-risk m^6^A group on patients’ biology-related functions. GSEA showed that epithelial-mesenchymal transition, angiogenesis, Myc target V1, and other pathways were mainly enriched in the high-risk group (Fig. 3I), whereas bile acid metabolism, KRAS signaling DN, estrogen response late, and other pathways were significantly enriched in low-risk patients (Fig. 3J) (Table 3).

**Table 3:** GSEA analysis results

Name	Size	Enrichment Score	NES	P value	Leading edge
HALLMARK_EPITHELIAL_MESENCHYMAL_TRANSITION	193	0.690003	3.006838	2.12E− 33	tags = 56%, list = 10%, signal = 51%
HALLMARK_ANGIOGENESIS	34	0.773029	2.613681	3.62E− 09	tags = 44%, list = 5%, signal = 42%
HALLMARK_MYC_TARGETS_V1	187	0.535557	2.344755	8.78E− 16	tags = 48%, list = 19%, signal = 39%
HALLMARK_MYC_TARGETS_V2	54	0.60481	2.295545	1.48E− 06	tags = 59%, list = 16%, signal = 50%
HALLMARK_ESTROGEN_RESPONSE_EARLY	187	-0.4833	− 1.50217	0.000929	tags = 33%, list = 12%, signal = 29%
HALLMARK_ESTROGEN_RESPONSE_LATE	188	-0.49748	− 1.54745	0.000127	tags = 43%, list = 16%, signal = 36%
HALLMARK_KRAS_SIGNALING_DN	191	-0.51553	− 1.60538	1.91E− 05	tags = 29%, list = 15%, signal = 25%
HALLMARK_BILE_ACID_METABOLISM	111	-0.53704	− 1.61638	0.000452	tags = 41%, list = 21%, signal = 32%

### Influence of m^6^A risk score on immune characteristics of patients with CESC

**Fig. 4:**
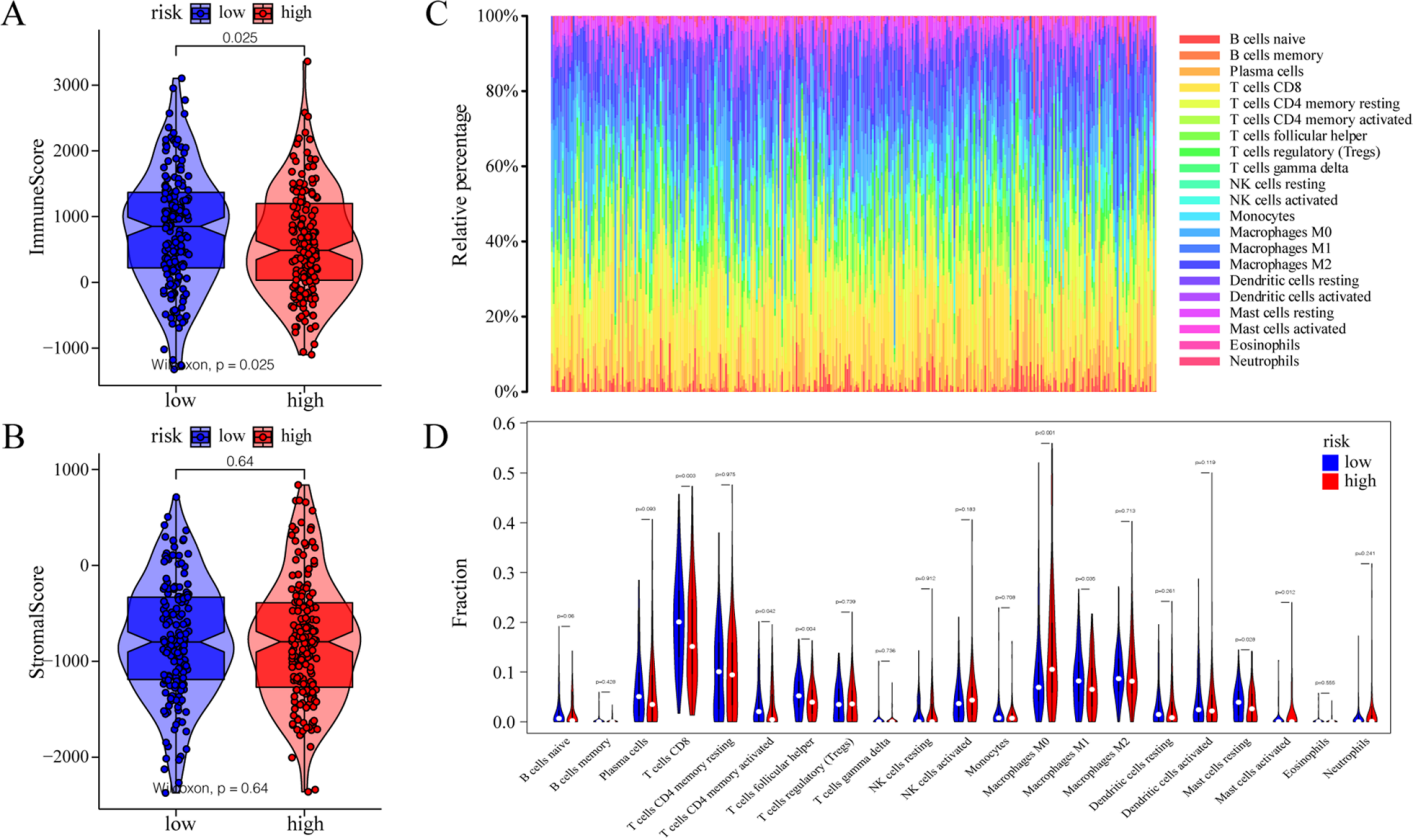
Association between m^6^A risk score and different immune cell infiltration. **A** Compared with the low expression group, the immune score was significantly lower in the high-risk group (P = 0.025). **B** There was no significant change in the stromal score in the high-risk group compared with that in the low-risk expression group (P = 0.64). **C** The CIBERSORT algorithm was used to analyze the overall level of immune cell infiltration in patients with CESC. **D** Correlation analysis showed significant differences in the expression of multiple immune cell subtypes between the high- and low-risk groups

Next, we assessed the impact of the m^6^A risk score on the overall immune characteristics and different levels of immune cell infiltration in patients with CESC. The immune score was significantly lower in the high-risk group than in the low-risk group (P = 0.025, Fig. 4A), whereas the stromal score was not significantly different (P = 0.64, Fig. 4B). Meanwhile, the CIBERSORT algorithm was further used to evaluate the invasion levels of 22 different immune cells (Fig. 4C). The difference analysis showed that the infiltration levels of various immune cell subsets differed significantly between the high- and low-risk groups (Fig. 4D), including T cells CD8, T cells, CD4 memory activated T cells, follicular helper cells, and M1 macrophages.

### Influence of m^6^A risk score on genomic changes in patients with CESC

**Fig. 5:**
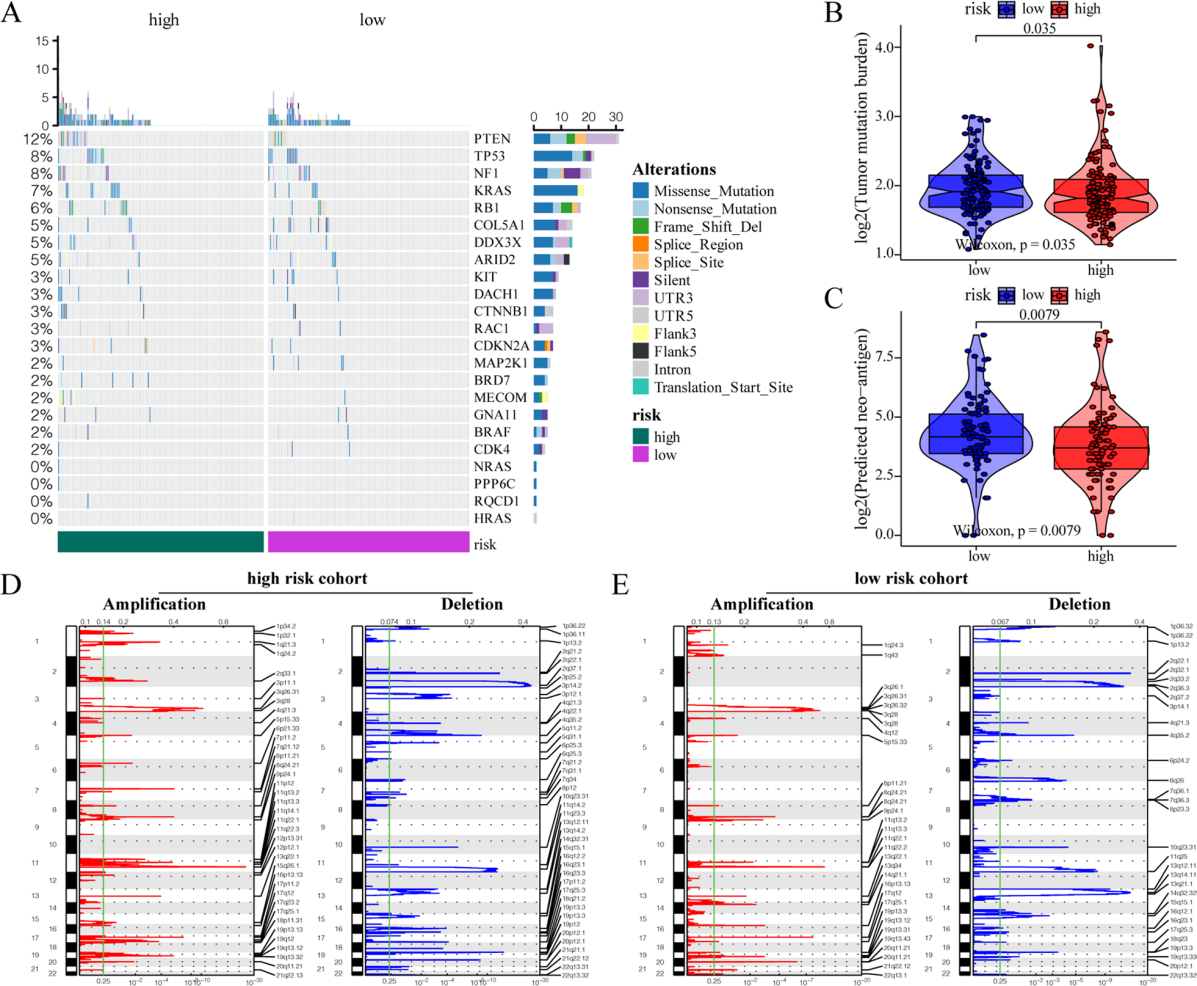
Influence of different m^6^A risk groups on genetic variation of patients with CESC. **A** Mutation profiles of common tumorigenesis drivers in the high- and low-risk patient groups. In the waterfall diagram, mutation information of each gene in each sample is displayed, and various colors indicate different mutation types. The section above the legend shows the mutation load. **B** The tumor mutation levels were significantly reduced in high-risk patients compared with those in low-risk patients (P = 0.035). **C** Tumor neoantigen levels were significantly lower in high-risk patients than in low-risk patients (P = 0.0079). **D**, **E** Changes in gene copy number levels in the high- and low-risk groups, where red represents genes with significantly increased copy numbers and blue represents genes with significantly missing copy numbers

Subsequently, we further assessed the impact of the m^6^A risk score on changes in levels of genetic variation in patients with CESC, including single nucleotide polymorphism and CNV. Analysis of single nucleotide mutations in driver genes during common tumorigenesis revealed that SNP levels in different driver genes were different between the high- and low-risk subgroups (Fig. 5A). Simultaneously, the overall level analysis showed that the TMB (P = 0.035, Fig. 5B) and tumor neoantigen levels (P = 0.0079, Fig. 5C) significantly differed between the high- and low-risk groups. Moreover, studies on the change frequency of CNV showed that CNV changes in high-risk patients were mainly focused on gene amplification (Fig. 5D), whereas in low-risk patients, CNV changes were mainly manifested as gene copy number deletion (Fig. 5E).

### Sensitivity analysis of patients with CESC to different small molecule drugs based on m^6^A risk score

**Fig. 6:**
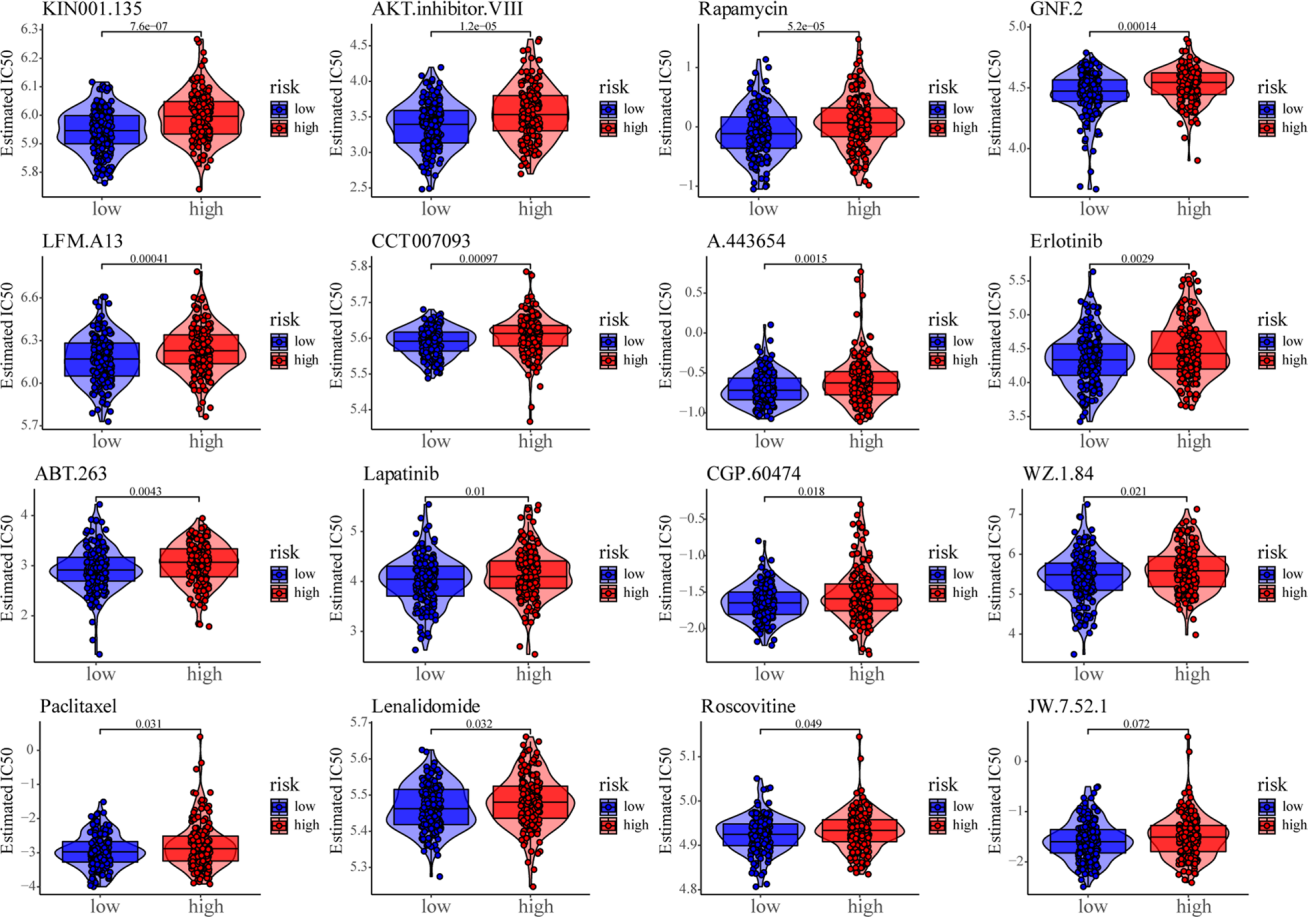
Sensitivity of the m^6^A risk score to different chemotherapy drugs and small molecule anticancer drugs was analyzed based on the GDSC database

To analyze the sensitivity of the m^6^A risk score to different drugs and small molecule substances in patients, we downloaded the cell line gene mutation data and IC_50_ values of different anticancer drugs from the GDSC database. IC_50_ values for patients with CESC were predicted based on the reactivity of cell lines to 138 different chemotherapeutic agents and small molecule anticancer agents. The results showed that the IC_50_ values of multiple chemotherapeutic agents and small molecule anticancer agents significantly differed between patients with high and low m^6^A risk scores (P < 0.001; Fig. 6), especially, KIN001.135, Akt.inhibitor.viii, and rapamycin.

### Construction of the clinical prediction model based on m^6^A risk score

**Fig. 7:**
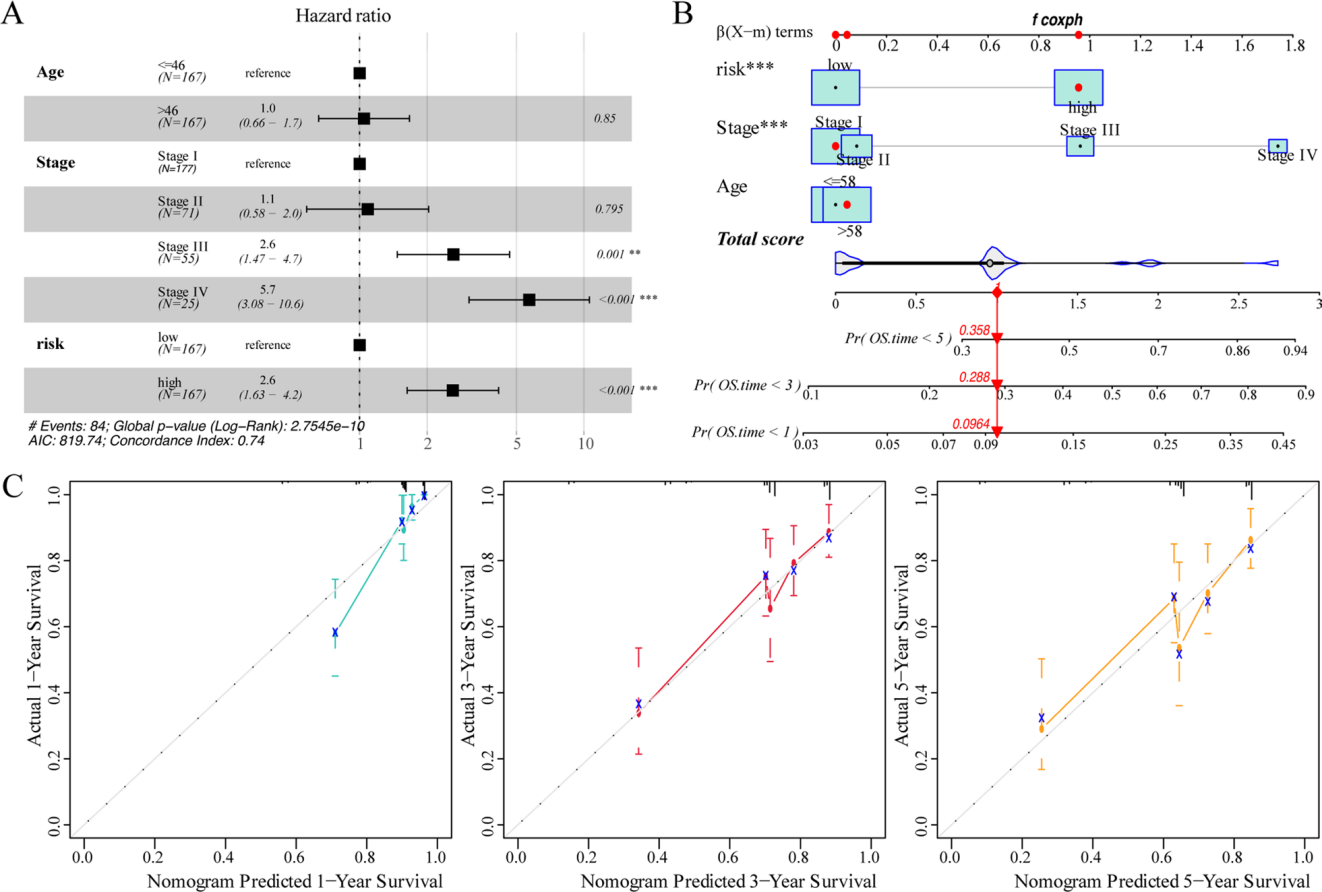
Prediction ability of the m^6^A risk score for patients with CESC. **A** Multivariate Cox regression analysis of HR and P-values of risk score combined with clinicopathological features. Analysis showed that the risk score was an independent risk factor for the prognosis of patients with CESC. **B** m^6^A risk scores coupled with clinicopathological features were selected to construct a clinical prediction model. **C** Calibration curve of the line diagram. The abscissa is the survival predicted by the line graph, and the ordinate is the actual observed survival, which was repeated 1000 times. The curve shows that the model has a good prognostic value for patients at 1, 3, and 5 years

Subsequently, we assessed the impact of the m^6^A risk score on the outcome of patients with CESC. Univariate and multivariate Cox analysis showed that the m^6^A risk score was an independent risk factor for predicting the prognosis of patients with CESC (Fig. 7A). Subsequently, the m^6^A risk score was combined with different clinicopathological features to construct a predictive rosette to predict the OS of patients with CESC (Fig. 7B). The C-index was used to calculate the distinguishing ability of the lipogram, which showed a high degree of differentiation [0.736 (0.680–0.792)]. Moreover, the calibration curves showed a good agreement between the 1-, 3-, and 5-year OS estimates from the rosettes and the actual patient observations (Fig. 7C).

### ZC3H13 expression in tissues

**Fig. 8:**
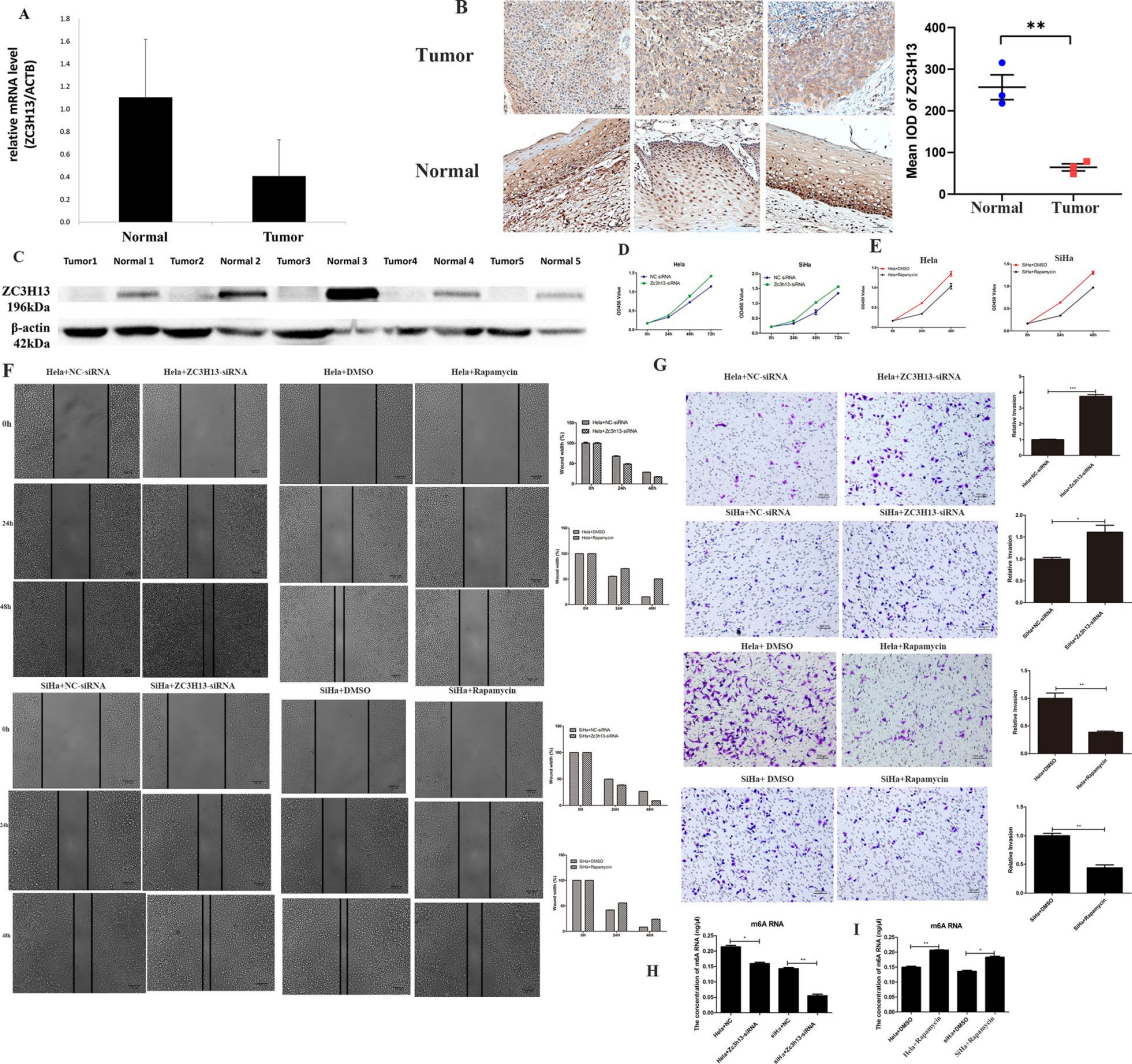
Expression and function of ZC3H13 in cervical cancer and the effects of rapamycin on cell phenotypes. **A** The qPCR analyses showed that ZC3H13 was significantly downregulated in cervical cancer tissues (P = 0.0019). **B** The IHC analysis showed that ZC3H13 was localized in the nucleus and was expressed at low levels in cervical cancer tissues (× 20) (scale bar: 50 μm). Gray level difference analysis showed that the IOD of ZC3H13 in normal cervical tissues was significantly higher than that in cervical cancer tissues. **C** The WB analysis demonstrated the low expression of ZC3H13 in cervical cancer tissues. **D** The CCK8 assay showed that the proliferation ability of HeLa and SiHa cells was significantly enhanced after ZC3H13 knockdown. **E** The CCK8 assay showed that the proliferation ability of HeLa and SiHa cells was significantly reduced after rapamycin treatment. **F** The wound-healing assay showed that ZC3H13 knockdown significantly enhanced the invasion abilities of the HeLa and SiHa cells. The wound-healing assay showed that the invasion ability of HeLa and SiHa cells was significantly reduced after rapamycin treatment. **G** The Transwell experiments showed that ZC3H13 knockdown significantly enhanced the migration abilities of the cells. The Transwell assay showed that the migration ability of HeLa and SiHa cells was significantly reduced after rapamycin treatment. **H** The m^6^A ELISA analyses showed that the m^6^A levels decreased significantly after the ZC3H13 knockdown. **I** The m^6^A ELISA analyses showed that the m^6^A levels increased significantly in both HeLa and SiHa cells after rapamycin treatment. All experiments were repeated at least three times (*P < 0.05, **P < 0.01, ***P < 0.001)

We used qPCR to study ZC3H13 expression in ten normal cervical tissues and ten cervical cancer tissues and found that ZC3H13 was significantly downregulated in cervical cancer tissues (Fig. 8A). The IHC analysis showed that ZC3H13 was localized in the nucleus and had a low expression in cervical cancer tissues (Fig. 8B). We performed gray level difference analysis of IHC results using the Image-Pro Plus software. The results showed that the mean integral optical density (IOD) of ZC3H13 in normal cervical tissues was significantly higher than that of ZC3H13 in cervical cancer tissues. The WB analysis also demonstrated the low expression of ZC3H13 in cervical cancer tissues (Fig. 8C).

### Function of ZC3H13 in cervical cancer and effects of rapamycin on cell phenotypes

The CCK8 assay showed that the proliferation of HeLa and SiHa cells was significantly enhanced after the ZC3H13 knockdown (Fig. 8D). Further, the CCK8 assay showed a significant reduction in the proliferation of HeLa and SiHa cells after rapamycin treatment (Fig. 8E). The wound-healing assay showed that ZC3H13 knockdown significantly enhanced the invasion ability of HeLa and SiHa cells (Fig. 8F). Transwell experiments showed that ZC3H13 knockdown significantly enhanced the cell migration ability (Fig. 8G). The wound-healing assay showed a significant reduction in the invasion abilities of HeLa and SiHa cells after rapamycin treatment (Fig. 8F). The Transwell assay showed a significant reduction in the migration abilities of HeLa and SiHa cells after rapamycin treatment (Fig. 8G). The m^6^A ELISA showed that the m^6^A levels decreased significantly after the *ZC3H13* knockdown (Fig. 8H) and that the m^6^A levels increased significantly in both HeLa and SiHa cells after rapamycin treatment (Fig. 8I).

## Discussion

As one of the most common RNA modifications, m^6^A mRNA methylation is closely associated with cervical cancer. m^6^A mRNA methylation might promote cervical cancer development. The m^6^A level was significantly reduced in cervical cancer compared to adjacent normal tissue. The reduction in m^6^A levels significantly correlated with the FIGO stage, tumor size, differentiation, lymph invasion, and cancer recurrence [38]. m^6^A methyltransferase methyltransferase-like 3 (METTL3) enhanced the stability of FOXD2-AS1, and its expression was maintained. METTL3/FOXD2-AS1 accelerated cervical cancer progression via an m^6^A-dependent modality [39]. METTL3 enhanced the HK2 stability through YTHDF1-mediated m^6^A modification, thereby promoting the Warburg effect in cervical cancer [40]. GAS5-AS1 interacted with the tumor suppressor GAS5 and increased its stability by interacting with RNA demethylase ALKBH5 and decreasing GAS5 m^6^A modification. m^6^A-mediated GAS5 RNA degradation relied on the m^6^A reader protein YTHDF2-dependent pathway [41]. FTO can control the m^6^A modification of E2F1 and Myc transcripts to regulate the proliferation and migration of cervical cancer cells [42]. The FTO-mediated stabilization of HOXC13-AS epigenetically upregulated FZD6 and activated Wnt/β-catenin signaling, driving CC proliferation, invasion, and EMT [43]. FTO and its substrate m^6^A may be critical factors for regulating chemo-radiotherapy resistance [44]. YTHDF1 regulated the translation of RANBP2, which potentiated the growth, migration, and invasion of cervical cancer cells in an m^6^A-dependent manner without any effect on its mRNA expression [45]. circARHGAP12 exerted the oncogenic role in cervical cancer progression through the m^6^A-dependent IGF2BP2/FOXM1 pathway [46]. KCNMB2-AS1 and IGF2BP3 formed a positive regulatory circuit that increased the tumorigenic effect of KCNMB2-AS1 in cervical cancer [47]. m^6^A-associated downregulation of miR-193b promoted cervical cancer aggressiveness by targeting CCND1 [48]. ZFAS1 and its m^6^A modification may be a promising target for cervical cancer treatment [49].

According to our study, differential analysis results showed differences in the expression of multiple m^6^A-related genes between cervical cancer tissues and normal tissues; furthermore, these genes were related to prognosis. The established m^6^A risk scoring system is closely related to the biological function of cervical cancer, immune invasion of cervical cancer, and sensitivity to small molecule drugs. The model based on the m^6^A signature genes and the model based on the m^6^A risk score can accurately predict the prognosis of patients with cervical cancer. Studies showed that m^6^A was closely related to epithelial–mesenchymal transition, angiogenesis, Myc targets V1, KRAS signal pathway, and estrogen in the occurrence and development of tumors [43, 50–54]. Our results showed that epithelial-mesenchymal transition, angiogenesis, Myc targets V1, and other pathways are mainly enriched in the high-risk groups, while bile acid metabolism, KRAS signaling DN, late estrogen response, and other pathways are significantly enriched in low-risk patients. The results of the present study may help understand the relationship between these pathways and m^6^A, revealing their mechanism in cervical cancer occurrence and development. We also showed that the m^6^A regulator correlates with the survival and clinicopathological characteristics of patients with CESC. The m^6^A regulator-based prognostic signature may predict the prognosis of CESC [55].

According to our analysis, the IC_50_ of multiple chemotherapy drugs differed significantly between patients with high and low m^6^A risk scores. Meanwhile, previous studies have also shown a close relationship between m^6^A, mTOR pathway, and rapamycin. mTORC1 could stimulate oncogenic signaling and control anabolic cell growth via m^6^A [56, 57]. m^6^A has been reported to play a fundamental role in the function of the mTOR pathway in gastrointestinal cancer [58]. Reductions in the m^6^A levels increased the expression of mTORC2 in endometrial cancer [59]. The FTO protein was reported to participate in tumorigenesis via interactions with the target of the mammalian target protein Rapamycin (mTOR) [60]. METTL3 was shown to cause the activation of mTORC1 signaling and colorectal cancer development in an m^6^A-dependent manner [61]. Rapamycin may regulate the m^6^A levels and affect the biological characteristics of OSCC cells [62]. METTL3-high tumors showed more sensitivity to everolimus, a rapamycin analog, in gastric cancer [63].

Our study showed that knocking down ZC3H13, an m^6^A methyltransferase, can promote the proliferation, migration, and invasion abilities of cervical cancer cells and reduce the m^6^A levels. Rapamycin suppresses the proliferation, migration, and invasion abilities of cancer cells and can enhance the m^6^A levels, demonstrating the role of the m^6^A methylation modulator in the development of cervical cancer and the effectiveness of the drugs targeting the modulators of m^6^A methylation in treating cervical cancer.

In conclusion, our study shows that m^6^A regulatory factors are closely related to the occurrence, development, immune invasion, drug sensitivity, and prognosis of cervical cancer. Drugs targeting the factors regulating m^6^A offer good prospects for treating cervical cancer.

## Supplementary Information

**Additional file 1.** Table of demographic and clinical characteristics of patients with cervical cancer.

**Additional file 2.** DEGs between different risk models.

**Additional file 3.** Pathways of different groups.

## Data Availability

The datasets used or analyzed during the current study are available from the TCGA GDC website (https://portal.gdc.cancer.gov/).
