# Supplementary material for: Gene signatures, immune infiltration, and drug sensitivity based on a comprehensive analysis of m6a RNA methylation regulators in cervical cancer
Source: J Transl Med. 2022 Sep 4;20:385. doi: 10.1186/s12967-022-03600-7 (PMC9441061; doi:10.1186/s12967-022-03600-7)
Supplement: Supplementary file 1 — Additional file 1. Table of demographic and clinical characteristics of patients with cervical cancer. [file 12967_2022_3600_MOESM1_ESM.docx]

Demographic and clinical characteristics of cervical cancer patients

| **Characteristic** | **high** | **low** |
| --- | --- | --- |
| n | 167 | 167 |
| Survival stutus, n (%) |  |  |
| Alive | 112 (33.5%) | 138 (41.3%) |
| Death | 55 (16.5%) | 29 (8.7%) |
| Stage, n (%) |  |  |
| Stage I | 91 (27.7%) | 86 (26.2%) |
| Stage II | 33 (10.1%) | 38 (11.6%) |
| Stage III | 25 (7.6%) | 30 (9.1%) |
| Stage IV | 16 (4.9%) | 9 (2.7%) |
| Age, median (IQR) | 45 (36.5, 54.5) | 48 (41, 61) |
